# COVAX delivers its 1 billionth COVID-19 vaccine doseCOVID-19 pandemic triggers 25% increase in prevalence of anxiety and depression worldwide

**Published:** 2022-04

**2 MArch 2022** **-** In the first year of the COVID-19 pandemic, global prevalence of anxiety and depression increased by a massive 25%, according to a scientific brief released by the World Health Organization (WHO) today. The brief also highlights who has been most affected and summarizes the effect of the pandemic on the availability of mental health services and how this has changed during the pandemic.

Concerns about potential increases in mental health conditions had already prompted 90% of countries surveyed to include mental health and psychosocial support in their COVID-19 response plans, but major gaps and concerns remain.

“The information we have now about the impact of COVID-19 on the world’s mental health is just the tip of the iceberg,” said Dr Tedros Adhanom Ghebreyesus, WHO Director-General. “This is a wake-up call to all countries to pay more attention to mental health and do a better job of supporting their populations’ mental health.”

## Multiple stress factors

One major explanation for the increase is the unprecedented stress caused by the social isolation resulting from the pandemic. Linked to this were constraints on people’s ability to work, seek support from loved ones and engage in their communities.

Loneliness, fear of infection, suffering and death for oneself and for loved ones, grief after bereavement and financial worries have also all been cited as stressors leading to anxiety and depression. Among health workers, exhaustion has been a major trigger for suicidal thinking.

## Young people and women worst hit

The brief, which is informed by a comprehensive review of existing evidence about the impact of COVID-19 on mental health and mental health services, and includes estimates from the latest Global Burden of Disease study, shows that the pandemic has affected the mental health of young people and that they are disproportionally at risk of suicidal and self-harming behaviours. It also indicates that women have been more severely impacted than men and that people with pre-existing physical health conditions, such as asthma, cancer and heart disease, were more likely to develop symptoms of mental disorders.

Data suggests that people with pre-existing mental disorders do not appear to be disproportionately vulnerable to COVID-19 infection. Yet, when these people do become infected, they are more likely to suffer hospitalization, severe illness and death compared with people without mental disorders. People with more severe mental disorders, such as psychoses, and young people with mental disorders, are particularly at risk.

## Gaps in care

This increase in the prevalence of mental health problems has coincided with severe disruptions to mental health services, leaving huge gaps in care for those who need it most. For much of the pandemic, services for mental, neurological and substance use conditions were the most disrupted among all essential health services reported by WHO Member States. Many countries also reported major disruptions in life-saving services for mental health, including for suicide prevention.

By the end of 2021 the situation had somewhat improved but today too many people remain unable to get the care and support they need for both pre-existing and newly developed mental health conditions.

Unable to access face-to-face care, many people have sought support online, signaling an urgent need to make reliable and effective digital tools available and easily accessible. However, developing and deploying digital interventions remains a major challenge in resource-limited countries and settings.

## WHO and country action

Since the early days of the pandemic, WHO and partners have worked to develop and disseminate resources in multiple languages and formats to help different groups cope with and respond to the mental health impacts of COVID-19. For example, WHO produced a story book for 6-11-year-olds, My Hero is You, now available in 142 languages and 61 multimedia adaptations, as well as a toolkit for supporting older adults available in 16 languages.

At the same time, the Organization has worked with partners, including other United Nations agencies, international nongovernmental organizations and the Red Cross and Red Crescent Societies, to lead an interagency mental health and psychosocial response to COVID-19. Throughout the pandemic, WHO has also worked to promote the integration of mental health and psychosocial support across and within all aspects of the global response.

WHO Member States have recognized the impact of COVID-19 on mental health and are taking action. WHO’s most recent pulse survey on continuity of essential health services indicated that 90% of countries are working to provide mental health and psychosocial support to COVID-19 patients and responders alike. Moreover, at last year’s World Health Assembly, countries emphasized the need to develop and strengthen mental health and psychosocial support services as part of strengthening preparedness, response and resilience to COVID-19 and future public health emergencies. They adopted the updated Comprehensive Mental Health Action Plan 2013-2030, which includes an indicator on preparedness for mental health and psychosocial support in public health emergencies.

## Step up investment

However, this commitment to mental health needs to be accompanied by a global step up in investment. Unfortunately, the situation underscores a chronic global shortage of mental health resources that continues today. WHO’s most recent Mental Health Atlas showed that in 2020, governments worldwide spent on average just over 2% of their health budgets on mental health and many low-income countries reported having fewer than 1 mental health worker per 100 000 people.

Dévora Kestel, Director of the Department of Mental Health and Substance Use at WHO, sums up the situation: ”While the pandemic has generated interest in and concern for mental health, it has also revealed historical under-investment in mental health services. Countries must act urgently to ensure that mental health support is available to all.”

Available from: https://www.who.int/news/item/02-03-2022-covid-19-pandemic-triggers-25-increase-in-prevalence-of-anxiety-and-depression-worldwide

